# A Computational Framework for Predicting Direct Contacts and Substructures within Protein Complexes

**DOI:** 10.3390/biom9110656

**Published:** 2019-10-25

**Authors:** Suyu Mei, Kun Zhang

**Affiliations:** 1 Software College, Shenyang Normal University, Shenyang 110034, China; 2 Bioinformatics Core of Xavier RCMI Center for Cancer Research, Department of Computer Science, Xavier University of Louisiana, New Orleans, LA 70125, USA

**Keywords:** protein complexes, complex substructure, machine learning, l_2_-regularized logistic regression, graph clustering, functional clustering

## Abstract

Understanding the physical arrangement of subunits within protein complexes potentially provides valuable clues about how the subunits work together and how the complexes function. The majority of recent research focuses on identifying protein complexes as a whole and seldom studies the inner structures within complexes. In this study, we propose a computational framework to predict direct contacts and substructures within protein complexes. In this framework, we first train a supervised learning model of l_2_-regularized logistic regression to learn the patterns of direct and indirect interactions within complexes, from where physical subunit interaction networks are predicted. Then, to infer substructures within complexes, we apply a graph clustering method (i.e., maximum modularity clustering (MMC)) and a gene ontology (GO) semantic similarity based functional clustering on partially- and fully-connected networks, respectively. Computational results show that the proposed framework achieves fairly good performance of cross validation and independent test in terms of detecting direct contacts between subunits. Functional analyses further demonstrate the rationality of partitioning the subunits into substructures via the MMC algorithm and functional clustering.

## 1. Introduction

Protein complexes have their individual gene products spatiotemporally arranged in place to form the structures required for specific biological activities [1]. Systematically investigating the disorder of subunits within protein complexes is crucial to elucidate the underlying mechanisms of various diseases [2]. In recent years, the majority of research, including experimental and computational methods, focuses on identifying protein complexes as a whole. For instance, the experimental techniques, e.g., tandem affinity purification with mass spectrometry (TAP-MS) and co-fractionation mass spectrometry (CF-MS), have been frequently used to detect protein complexes. In addition, many computational methods have been proposed to rapidly provide global landscape of genome-scale protein complexes. The well-known databases of protein complexes include MIPS [3], CORUM [4], HPRD [5] and Reactome [6,7]. MIPS [3] collects the protein complexes of *Saccharomyces cerevisiae*. CORUM [4] is a public repository of experimentally-characterized protein complexes from mammalian organisms. HPRD [5] provides a set of experimentally verified protein complexes from *Homo sapiens*. Reactome [6,7] provides a large number of co-complexed protein pairs from *Homo sapiens*. Interested readers can refer to References [8,9] for comprehensive surveys of the experimental techniques, computational methods and databases. However, most of these studies seldom explore the hierarchical substructures and interactions between subunits within complexes.

To our knowledge, there are only several studies that investigate the inner structure of complexes. For instance, Gavin et al. [1] propose a socio-affinity index to partition proteins in complexes into core components and attachments. Aloy et al. [10] provide a fine resolution to the interactions between subunits within complexes via homology modeling and electron microscopy. However, both methods could not identify the direct contacts between subunits. Drew et al. [11] propose a sparse graphical model learning framework to predict physical interactions from CF-MS data via covariation pattern of protein abundances, and then map the physical interactions into protein complexes to infer the substructures. Friedel et al. [12] derive scored protein–protein interaction (PPI) networks from TAP-MS data, on which the calculation of maximum spanning trees (MST) for physical interaction prediction is based. The MST is further partitioned into disjoint sub-complexes according to the weights of interactions.

To gain knowledge about inner substructures of protein complexes, we systematically designed a two-step computational model. The first step was to predict the physical interactions between subunits within complexes, and the second step was to partition the subunit interaction networks into sub-complexes via graph clustering. In the first step, we needed to cautiously restrict the physical interactions within complexes (called complex-scale physical interactions) rather than between genome-scale individual proteins (called interactome-scale physical interactions). Both methods [11,12] predict direct protein interactions from global-view CF-MS proteomic data and further infer the inner substructures within protein complexes. To date, most of the existing computational methods focus on predicting interactome-scale physical interactions [13,14,15], and no computational methods have been proposed to predict physical subunit interactions within complexes. From a computational point of view, these two problems are distinct in the modeling process. First, complex-scale interactions are more restricted within complexes while interactome-scale interactions take place in the same organelle among genome-scale proteins. The patterns of physical interactions are potentially different. Secondly, the subunits within complexes basically assume indirect interactions though not direct contact, while the genome-scale proteins that do not physically interact are probably not to be functionally associated at all. As such, the modeling process for complex-scale physical interactions is quite different in terms of constructing training data.

The second step was to infer sub-complexes from the predicted networks of physical subunit interactions within complexes. Drew et al. [11] calculate the conditionally dependent PPIs to predict direct contacts and group the directly-contacted subunits into sub-complexes. Friedel et al. [12] use the weights of maximum spanning trees (MST) to cluster proteins into disjoint sub-complexes. Actually, the sub-complexes are potentially hierarchically-organized and overlapped. For super-complexes with a large number of subunits, we needed to resort to sophisticated graph clustering methods to gain fine-grained resolution of the inner structures of complexes. Recently, many graph clustering algorithms have been proposed to identify protein complexes from genome-scale PPI networks or CF-MS proteomic data [8,9]. For instance, the well-accepted Markov clustering (MCL) method [16] simulates random walks on PPI networks via expansion and inflation operators to extract dense regions as protein complexes. Different from these methods, we attempted to conduct graph clustering on the physical subunit interaction networks within complexes.

In this study, we propose a computational framework that combined supervised learning and graph clustering to predict physical subunit interactions and infer substructures within human protein complexes. The direct and indirect interactions of training data were restricted within complexes. A graph clustering method, named maximum modularity clustering (MMC) [17], was used to infer inner substructures from the predicted physical subunit interaction networks within complexes. As shown by Noack et.al [17], MMC demonstrates good performance in inferring hierarchically-organized and overlapped clusters. For fully-connected networks of physical subunit interaction, we used a functional clustering method to infer sub-complexes.

## 2. Materials and Methods

### 2.1. Flowchart of the Proposed Framework

**Figure 1:**
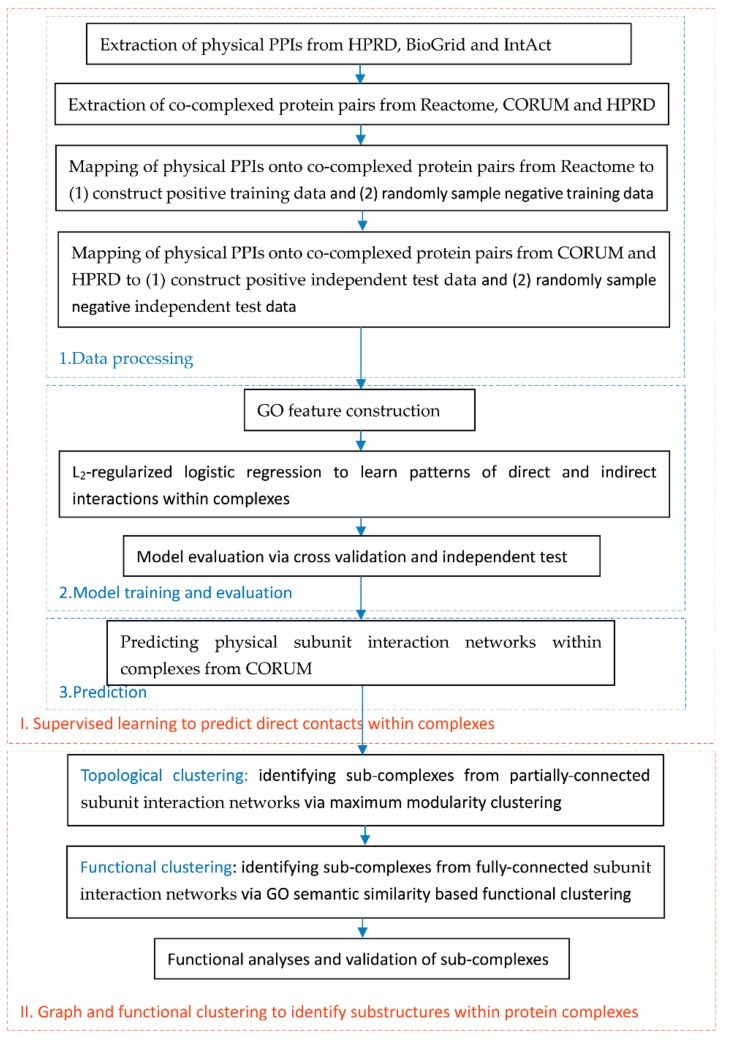
Flowchart of the proposed framework.

We first show the flowchart of the proposed framework for easy grasp of the workflow. As illustrated in Figure 1, this study was divided into two major phases. The first phase was to build a supervised learning model to predict direct contacts within complexes, and the second phase was to identify substructures via graph clustering from the predicted physical subunit interaction networks. The first phase consisted of three steps. Firstly, to construct the positive data for training and independent test sets, we mapped the physical PPIs from HPRD [5], BioGrid [18] and IntAct [19] onto the co-complexed protein pairs from Reactome [6,7], CORUM [4] and HPRD [5]. The negative training data and independent test data were randomly sampled from the indirect interactions within complexes; secondly, each gene pair was represented with a gene ontology (GO) feature vector to train a supervised learning model and the model was estimated via cross validation and independent test. Lastly, we used the trained model to predict the physical subunit interaction networks within complexes from CORUM [4].

In the second phase, we conducted clustering on the predicted subunit interaction networks to identify sub-complexes. For partially-connected networks, we used topological clustering via maximum modularity clustering (MMC) [17]; and for fully-connected networks, we used functional clustering to group genes based on GO semantic similarities.

### 2.2. Construction of Training and Independent Test Data

#### 2.2.1. Positive Training and Independent Test Data

To learn the patterns of direct and indirect subunit interaction, we restricted the construction of training data within complexes. We first obtained physical protein–protein interactions from HPRD [5], BioGrid [18] and IntAct [19]. After filtering out the proteins that were obsolete, uncurated or had no gene names, we obtained 57,920 non-redundant physical PPIs in total. These interactome-scale PPIs covered genome-scale genes and thus could not be used to predict complex-scale subunit interactions within complexes. We obtained 50,550 co-complexed protein pairs including 163 indirect interactions from Reactome [6,7]. We mapped the 57,920 physical PPIs onto the 50,550 co-complexed pairs and filtered out the 163 indirect interactions to obtain 9125 co-complexed physical PPIs as the positive training data (see Supplementary File S1).

We further mapped the 57,920 physical PPIs to the co-complexed protein pairs from CORUM [4] and HPRD [5], and obtained 3326 and 2349 co-complexed physical PPIs respectively as the positive independent test data. We ensured that there was no overlap between the positive independent test data and the positive training data. During data processing, only well-studied genes were kept with the less-studied genes discarded because less-studied genes would result in a null feature vector (see the subsection “Feature Construction”). Well-studied genes refer to the genes annotated with at least one GO term of molecular function of biological process.

#### 2.2.2. Negative Training and Independent Test Data

In the proposed framework, the negative class refers to indirect subunit interactions within complexes. Besides the 163 indirect interactions from Reactome [6,7], we needed to further sample a large number of negative training data so that the two classes were of equal size, i.e., 9125 co-complexed indirect interactions. The remaining negative data were sampled in the space of co-complexed protein pairs from CORUM [4] and HPED [5]. To obtain credible indirect interactions, we imposed a constraint that the path length of the shortest paths, if any, between two co-complexed proteins was no less than two (referred to as No-less-than-two). If no path existed between two co-complexed proteins, the path length was assumed to infinity (∞) (referred to as No-path). In general, the indirect interactions sampled in the No-path case were more credible than those sampled in the No-less-than-two case. We introduced a ratio λ of No-path case to No-less-than-two case to balance the sampling of negative data. The negative training data contained 9125 co-complexed indirect interactions (see Supplementary File S2).

Two negative independent test sets containing 3326 and 2349 co-complexed indirect interactions were similarly sampled from CORUM [4] and HPED [5], respectively. In addition, the indirect interactions from KEGG [20] were used as the third negative independent test set, which contained only four indirect interactions after filtering out the overlap with the other positive independent test sets.

### 2.3. Supervised Learning for Predicting Direct Contacts within Protein Complexes

#### 2.3.1. Feature Construction

Gene ontology (GO) has been reported to be the most discriminative feature to depict protein pairs and predict PPIs [21]. Unfortunately, GO knowledge is highly imbalanced among genes. To address the issues about sparsity and potential unavailability of GO terms for less-studied genes/proteins, homolog GO knowledge was transferred to enrich the genes/proteins concerned, so that each protein pair was depicted with two instances, i.e., target instance and homolog instance. The target instance depicted the GO knowledge of the gene/protein itself and the homolog instance depicted the GO knowledge of the homologs. When a gene/protein was completely not annotated, the homolog instance could be used as a substitute. We ran PSI-BLast [22] against all species in SwissProt [23] to obtain homologs and extract the GO terms for each gene/protein from GOA [24].

For each protein *i* in the training set *U*, we obtained the homolog set of GO terms denoted as $G_{{}_{H}}^{i}$ and the target set of GO terms denoted as $G_{{}_{T}}^{i}$. The entire set of GO terms *G* is defined as follows.

(1) $$G=\underset{i\in U}{\cup }(G_{{}_{T}}^{i}\cup G_{{}_{H}}^{i}).$$

The feature vectors for target instance and homolog instance for protein pair $\left(i_{1},i_{2}\right)$ are formally defined as follows.

(2) $$R_{T}^{\left(i_{1},i_{2}\right)}[g]=\left\{\begin{array}{l} 0,g\notin G_{T}^{i_{1}}\wedge g\notin G_{T}^{i_{2}}\\ 2,g\in G_{T}^{i_{1}}\wedge g\in G_{T}^{i_{2}}\\ 1,otherwise \end{array}\right.;\;\;R_{H}^{\left(i_{1},i_{2}\right)}[g]=\left\{\begin{array}{l} 0,g\notin G_{H}^{i_{1}}\wedge g\notin G_{H}^{i_{2}}\\ 2,g\in G_{H}^{i_{1}}\wedge g\in G_{H}^{i_{2}}\\ 1,otherwise \end{array}\right..\;$$

For a GO term g∈G, $R_{T}^{\left(i_{1},i_{2}\right)}[g]$ and $R_{H}^{\left(i_{1},i_{2}\right)}[g]$ denote the component *g* of feature vector for the target instance and homolog instance, respectively. The GO terms g∉G are discarded. If protein pair $\left(i_{1},i_{2}\right)$ share a common *GO* term *g*, the value of component *g* in both feature vector is set to 2; if neither protein possesses the *GO* term *g*, the value is set to 0; otherwise the value is set to 1. This simple method of feature representation intuitively represents the distribution of GO terms among two proteins without considering the hierarchical and semantic relationship between GO terms. As compared with the method that incorporates the ancestor GO terms in GO directed acyclic graph (DAG) [21], this simple method can reduce the inter-feature correlations. GO semantic similarities are more appropriate to be embedded into the kernel method [25]. Due to sparsity of GO terms, dimensionality reduction was also not applicable to GO feature representation.

#### 2.3.2. Supervised Learning via L_2_-Regularized Logistic Regression

Computational complexity and noise tolerance were two major concerns for us to choose the base classifier. Since a regularization technique can counteract the noise from homolog knowledge transfer and logistic regression performs well in fast training of large-scale data, we selected the well-established l_2_-regularized logistic regression method [26] that is implemented in the toolbox LIBLINEAR [27] as the classifier. In the training phase, the target and homolog instance of a protein pair both participated in the model training. Given training data *x* and labels *y* that consist of a set of instance-label pairs $\left(x_{i},y_{i}),i=1,2,\ldots ,l;x_{i}\in R^{n};y_{i}\in \{-1,+1\right\}$, the decision function of logistic regression is defined as $F(x)=1/\left(1+\exp(-y\omega^{T}x)\right)$. L_2_-regularized logistic regression calculates the optimum weight vector ω via solving the following optimization problem.

(3) $$\underset{\omega }{\min}\frac{1}{2}\omega^{T}\omega +C\sum_{i=1}^{l}\log(1+e^{-y_{i}\omega^{T}x_{i}}),$$

where C denotes the penalty parameter or regularizer. The second term penalizes potential noise/outlier fitting. The prime optimization problem as defined by Equation (3) is solved via its dual form as follows.

(4) $$\begin{array}{l} \underset{\alpha }{\min}\frac{1}{2}\alpha^{T}Q\alpha +\sum_{i:\alpha_{i}>0}^{l}\alpha_{i}\log\alpha_{i}+\sum_{i:\alpha_{i}<C}\left(C-\alpha_{i}\right)\log(C-\alpha_{i})-\sum_{i}^{l}C\log C\\ subject\;to\;\leq \alpha_{i}\leq C,i=1,\ldots ,l \end{array}$$

where $\alpha_{i}$ denotes the Lagrangian operator and $Q_{ij}=y_{i}y_{j}x_{i}^{T}x_{j}$.

In the test or prediction phase, the decision function F(x) yields two outputs $F(R_{T}^{\left(i_{1},i_{2}\right)}){,}_{}F(R_{H}^{\left(i_{1},i_{2}\right)})$ for each protein pair $\left(i_{1},i_{2}\right)$, which are combined into one final decision value as defined below.

(5) $$Decision\_value(i_{1},i_{2})=\left\{\begin{array}{l} F(R_{T}^{\left(i_{1},i_{2}\right)}),if_{}|F(R_{T}^{\left(i_{1},i_{2}\right)})|>|F(R_{H}^{\left(i_{1},i_{2}\right)})|\\ F(R_{H}^{\left(i_{1},i_{2}\right)}),otherwise \end{array}\right.,$$

where |•| denotes absolute value. The final label for the test protein pair $\left(i_{1},i_{2}\right)$ is defined as follows.

(6) $$L(i_{1},i_{2})=\left\{\begin{array}{l} 1,Decision\_value(i_{1},i_{2})>0\wedge Decision\_value(i_{1},i_{2})-0.5>\delta \\ -1,Decision\_value(i_{1},i_{2})<0\wedge -Decision\_value(i_{1},i_{2})-0.5>\delta \\ \propto ,otherwise \end{array}\right.,$$

where the threshold δ is used to filter out those weak positive predictions, and ∝ denotes undetermined status that will be discarded.

### 2.4. Graph Clustering for Sub-Complexes Discovery

#### 2.4.1. Topological Clustering via Maximum Modularity Clustering

As illustrated in Figure 1, the second phase of the proposed framework was to cluster the physical subunit interaction networks into sub-complexes. For partially-connected networks, we used a graph clustering method to discover sub-complexes. It was noted that the two phases were related via the predicted networks of physical subunit interaction, which was the output of the first phase and the input of the second phase. Nevertheless, the computational methods adopted in the two phases were independent with the mathematical symbols valid within their own scopes. In this study, we used the maximum modularity clustering method (MMC) [17] to infer the inner substructures within complexes. This graph clustering method heuristically searches the optimal partitioning of a graph via iterative coarsening and refining operators. The coarsening operator merges clusters, while the refining operator iteratively moves individual vertices between the resulting clusters according to the criteria of modularity increase (MI).

Assuming that a graph (V,f) consists of a vertex set *V* and a function f:V×V→N, the function *f* assigns an edge weight to each vertex pair. The degree of vertex *v* is defined as $\deg(v)=\sum_{u\in V}f(u,v)$. The degree of a set of vertices is generalized as $\deg(V)=f(V,V)=\sum_{u\in V,v\in V}f(u,v)$. The aim of graph clustering is to partition the vertex set *V* into non-empty subsets $C=\{C_{1},\ldots ,C_{k}\}$ partitions. In the null model where the end-vertices of 1/2deg(V) edges are chosen at random, each vertex pair $(u,v)\in V^{2}$ has the edge weight f(u,v) binomially distributed and the expected value of edge weight is $\deg(u)\deg(v)/\deg{\left(V\right)}^{2}$. This conclusion could be generalized to an edge set [28]. The modularity of the clustering $C=\{C_{1},\ldots ,C_{k}\}$ is defined as follows.

(7) $$Q(C):=\sum_{C_{i}\in C}\left(f(C_{i},C_{i})/f(V,V)-dev{\left(C_{i}\right)}^{2}/dev{\left(V\right)}^{2}\right).$$

The first term is the actual fraction of intra-cluster edge weight and the second term specifies the expected fraction of intra-cluster edge weight in the null model. Then the modularity increase caused by the coarsening operator that merges cluster $C_{i}$ and $C_{j}$ is defined as follows.

(8) $$\Delta Q_{C_{i},C_{j}}:=2f(C_{i},C_{j})/f(V,V)-2dev(C_{i})dev(C_{j})/dev{\left(V\right)}^{2}.$$

The modularity increase caused by the refining operator that moves a vertex *v* from its cluster $C_{i}$ to another cluster $C_{j}$ is defined as follows.

(9) $$\Delta Q_{v->C_{j}}:=2(f(v,C_{j})-f(v,C_{i}-v))/f(V,V)-2(dev(v)dev(C_{j})-\deg(v)dev(C_{i}-v))/dev{\left(V\right)}^{2}.$$

The coarsening and refining operator iterate greedily until no modularity increase ($\Delta Q_{C_{i},C_{j}}<0$, $\Delta Q_{v->C_{j}}<0$) to achieve maximum modularity.

#### 2.4.2. Functional Clustering via GO Semantic Similarities

For fully-connected subunit interaction networks, the topological clustering method was no longer applicable. Instead, we employed functional clustering to understand the functional associations between subunits within complexes. In this study, we used GO semantic similarities to group closely associated subunits into sub-complexes. Wang et al. [29] proposed a measure called S-value to measure the semantic similarity between two GO terms. The S-value of a GO term is defined through its closest ancestor and children GO terms in GO directed acyclic graph (DAG). Then the S-values of common ancestor GO terms is used to define the semantic similarity between two GO terms. Given a GO term *A* and its $DAG_{A}=(A,T_{A},E_{A})$, where $T_{A}$ denotes the GO term set that includes *A* and its ancestor GO terms in GO DAG and $E_{A}$ denotes the set of edges, for any GO term $t\in DAG_{A}$, the S-value of *t* related to *A* is defined as below.

(10) $$S_{A}(t)=\left\{\begin{array}{l} 1,t=A\\ \max\{w_{e}\times S_{A}(t^{\prime })|t^{\prime }\in childrenof(t)\},t\neq A \end{array}\right.,$$

where $w_{e}$ denotes the weight of the edge linking term *t* to its child term $t^{\prime }$, assuming 0.8 and 0.6 for is-a and part-of relations, respectively. The semantic value of GO term *A* is defined as follows.

(11) $$SV(A)=\sum_{t\in A}S_{A}(t).$$

Based on Formulae (10,11), the semantic similarity between GO term *A* and *B* is defined below.

(12) $$S_{GO}(A,B)=\frac{\sum_{t\in T_{A}\cap T_{B}}\left(S_{A}(t)+S_{B}(t)\right)}{SV(A)+SV(B)}.$$

GO semantic similarities could be aggregated to define gene similarity via the methods of maximum, average and best-match average [29]. In this study, we only roughly gain knowledge of coarse-level modular organizations within complexes and hence we adopt the maximum strategy to calculate gene functional similarities. Given two genes $g_{1},g_{2}$ with GO term set $GO_{1}=\{go_{11},go_{12},\ldots ,go_{1m}\}$ and $GO_{2}=\{go_{21},go_{22},\ldots ,go_{2n}\}$, respectively, the functional similarity between $g_{1}$ and $g_{2}$ is calculated via max strategy as follows.

(13) $$Sim(g_{1},g_{2})=\underset{1\leq i\leq m,1\leq j\leq n}{\max}S_{GO}(go_{1i},go_{2j}).$$

### 2.5. Experimental Setting and Model Evaluation

#### 2.5.1. Supervised Learning

The l_2_-regularized logistic regression model is evaluated under three experimental settings, namely combined-instance, homolog-instance and target-instance. The combined-instance setting uses both target instances and homolog instances to evaluate the model; the homolog-instance setting uses homolog instances alone to evaluate whether the model is robust against GO unavailability; and the target-instance setting uses target instances to estimate the baseline performance. We adopted five performance metrics, i.e., receiver operating characteristic (ROC) and area under the curve (AUC) (ROC-AUC), precision (PR), specificity (SE), Matthews correlation coefficient (MCC) and F1 score. Among these metrics, PR, SE and MCC were derived from a confusion matrix *M*, where its element $M_{i,j}$ records the counts that class i are classified to class j. For the convenience of calculation, we first derived several intermediate variables from *M* via Formula (14). The we calculated the class-specific metrics PR_l_, SE_l_ and MCC_l_ via Formula (15). The overall MCC is calculated via Formula (16).

(14) $$\begin{array}{l} p_{l}=M_{l,l},q_{l}=\sum_{i=1,i\neq l}^{L}\sum_{j=1,j\neq l}^{L}M_{i,j},r_{l}=\sum_{i=1,i\neq l}^{L}M_{i,l},s_{l}=\sum_{j=1,j\neq l}^{L}M_{l,j}\\ p=\sum_{l=1}^{L}p_{l},q=\sum_{l=1}^{L}q_{l},r=\sum_{l=1}^{L}r_{l},s=\sum_{l=1}^{L}s_{l} \end{array}$$

(15) PRl=plpl+rl,l=1,2…,LSEl=plpl+sl,l=1,2…,LMCCl=(plql−rlsl)/(pl+rl)(pl+sl)(ql+rl)(ql+sl),l=1,2…,L

(16) $$\begin{array}{l} Acc=\sum_{l=1}^{L}M_{l,l}/\sum_{i=1}^{L}\sum_{j=1}^{L}M_{i,j}\\ MCC=\left(pq-rs\right)/\sqrt{\left(p+r)(p+s)(q+r)(q+s\right)} \end{array}$$

where *L* denotes the number of labels. For binary classification, *L* is equal to two. The AUC score was calculated on the basis of the decision values calculated via Formula (5). The F1 score is defined as follows:

(17) F1 score=2×PRl×SElPRl+SEl,l=1 denotes the positive class.

#### 2.5.2. Graph Clustering

To measure the performance of graph clustering, we used a Jaccard index to estimate how well the predicted set of sub-complexes *P* matchec the actual set of sub-complexes *C*.

(18) Jaccard(P,C)=|P∩C|/|P∪C|.

Given a threshold ξ, we deem *P* matches *C* if Jaccard(P,C)≥ξ is satisfied (ξ generally assumes 0.5). Accordingly, the metrics of precision, recall and F-score for graph clustering are defined as follows.

(19) Precision=|{Pi∈P|∃Cj∈C,Jaccard(Pi,Cj)≥ξ}||P|Recall=|{Ci∈C|∃Pj∈P,Jaccard(Pj,Ci)≥ξ}||C|F-score=2×Precision×RecallPrecision+Recall

## 3. Results

### 3.1. Performance of Predicting Physical Subunit Interactions within Complexes

As mentioned in the subsection “Negative training and independent test data”, the negative data were sampled from two sources: (1) the co-complexed protein pairs that no path existed between them in human physical PPI networks (No-path); and (2) the co-complexed protein pairs connected via paths whose path lengths all were no less than two (No-less-than-two). The sampling ratio λ between No-path and No-less-than-two was empirically determined. The computational results showed that the model achieved optimum performance of cross validation and independent test at the ratio λ=4 and the negative class is provided in Table 1.

**Figure 2:**
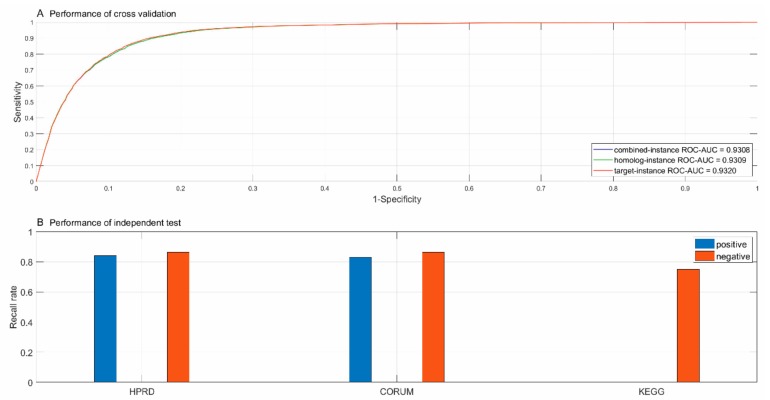
Performance of 5-fold cross validation (**A**) and independent test (**B**).

As illustrated in Figure 2A, the ROC curves of the three experimental settings nearly coincided and the proposed framework achieved fairly high AUC scores. The results showed that homolog knowledge transfer via homolog instances was effective and the model could work when the concerned genes/proteins were hardly annotated. As shown in Figure 2B, the proposed framework achieved satisfactory performance on the positive and negative independent test data from CORUM [4] and HPRD [5]. In addition, three out of four experimentally verified indirect interactions from KEGG [20] were validated by the proposed framework. The encouraging performance on the negative class showed that the negative data sampling strategy adopted by the proposed framework was rational and credible.

The performance measured via precision, sensitivity and MCC on the positive and negative class is provided in Table 1. The results showed that the proposed framework performed very well on both classes and showed low risk of bias the three experimental settings. The performance of cross validation and independent test showed that the proposed framework could satisfactorily identify physical subunit interactions and facilitate further inferring the inner substructures within complexes.

### 3.2. Inferring Substructures within Complexes from CORUM [4]

#### 3.2.1. Identifying Physical Interactions within Complexes from CORUM [4]

**Figure 3:**
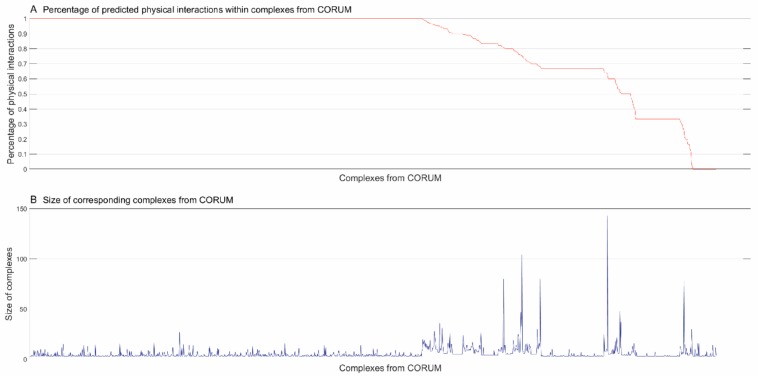
Percentage of predicted physical interactions within complexes in CORUM [4]. (**A**) The predicted connection degrees of complexes in CORUM [4] in descending order; (**B**) the actual size of corresponding complexes in CORUM [4].

We used the trained model to further predict the physical subunit interactions within complexes from CORUM [4]. After removing the complexes that contained fewer than three subunits, we totally obtained 1428 complexes from CORUM [4]. For a complex with *N* subunits, there are potentially maximum *N* × (*N* − 1)/2 physical interactions or edges, i.e., a complete graph. For a complex that is predicted to possess *M* physical subunit interactions, we define the connection degree as 2MN×(N−1) to measure the graph density. According to the computational results, 57.21% of complexes were predicted to have fully-connected subunits, 39.29% of complexes were predicted to have partially-connected subunits and the remaining 3.5% of complexes were predicted to have completely-isolated subunits. In Figure 3, the relationships between predicted connection degrees (A) and the size of complexes (B) are illustrated, wherein the horizontal axis denotes the complexes from CORUM [4] with the predicted connection degrees in descending order. From Figure 3A,B, we can see that the majority of predicted fully-connected complexes contained a small number of subunits and most of the large complexes were predicted to have fewer physical interactions. The 3.5% of complexes whose subunits were predicted to be fully isolated were potentially bridging or boundaries across complexes, or no physical interactions were predicted just because of false negative predictions.

#### 3.2.2. Inferring Substructures within Partially-Connected Complexes via Mmc Algorithm

**Figure 4:**
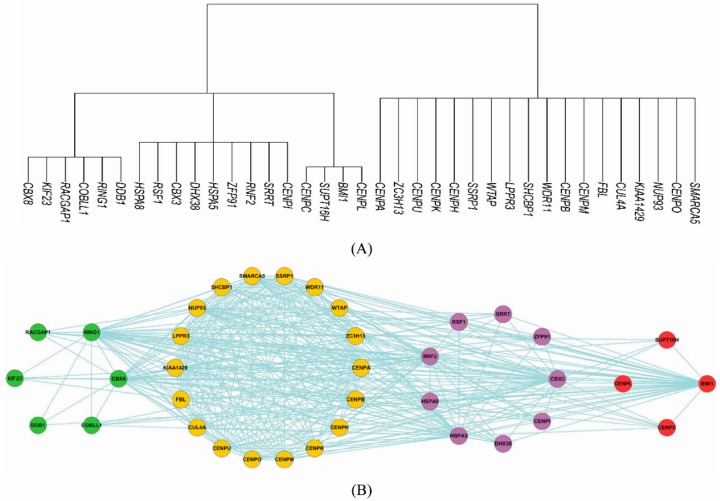
Inferred hierarchical sub-complexes via Matthews correlation coefficient (MCC) graph clustering (**A**) and the inner topological visualization (**B**) within centromere chromatin (CEN) complex from CORUM [4] (37 subunits).

For the complexes whose subunits are predicted to be partially connected, we used the maximum modularity clustering method (MMC) [17] to identify the modularity. As shown in Figure 3A, about 40% complexes potentially demonstrated inner topological modularity. Take centromere chromatin complex (CEN complex) for an example. The complex was composed of 37 subunits and was predicted to possess 50.60% connection degree. According to Schalch et al. [30], a centromere serves as the attachment site for microtubules to mediate chromosome segregation during mitosis and meiosis. The centromere core and its flanking pericentric heterochromatin form a structure that exposes CENP-A-containing chromatin to the surface to interact with the kinetochore and microtubules. In this study, the MMC method split the CEN complex into four clusters as shown in Figure 4A. As illustrated in Figure 4B, the inner topological visualization shows that the intra-cluster links were sparse while the inter-cluster links were dense, indicating potentially heavy signaling traffic between sub-complexes.

**Figure 5:**
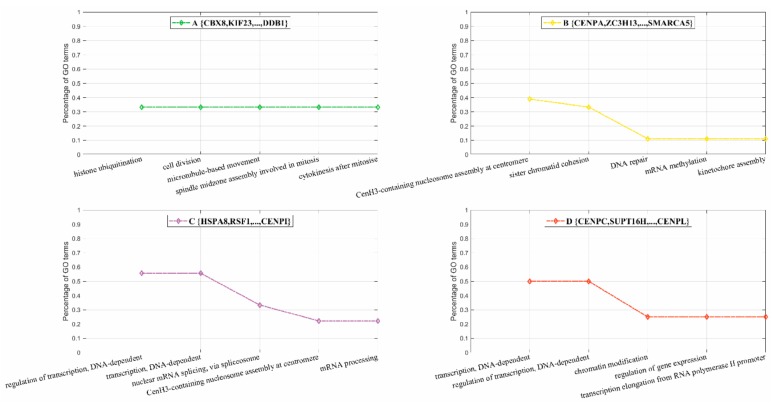
Gene ontology (GO) enrichment analyses of the sub-complexes (**A**) {CBX8, KIF23,…, DDB1}; (**B**) {CENPA, ZC3H13,…, SMARCA5}; (**C**) {HSPA8,RSF1,…,CENPI}; (**D**) {CENPC, SUPT16H, …, CENPL} within the CEN complex inferred via the MMC algorithm.

We further conducted functional GO enrichment analyses of the four sub-complexes within the CEN complex. Top five GO terms were provided for each sub-complex. As illustrated in Figure 5A, the sub-complex {CBX8, KIF23,..., DDB1}, corresponding to the nodes in green in Figure 4A, had its subunits majorly involved in the processes of cell division, e.g., GO:0051301 cell division; GO:0007018 microtubule-based movement; GO:0051256 spindle midzone assembly involved in mitosis, etc. As illustrated in Figure 5B, the sub-complex {CENPA, ZC3H13, ..., SMARCA5} had its subunits involved in centromere activity, e.g., GO:0034080 CenH3-containing nucleosome assembly at centromere; GO:0007062 sister chromatid cohesion; GO:0051382 kinetochore assembly, etc. As illustrated in Figure 5C,D, the other two sub-complexes had their subunits involved in the processes of regulation of transcription, e.g., GO:0006355 regulation of transcription, DNA-dependent; GO:0000398 nuclear mRNA splicing, via spliceosome; GO:0010468 regulation of gene expression, etc.

#### 3.2.3. Inferring Substructures within Fully-Connected Complexes via Functional Clustering

**Figure 6:**
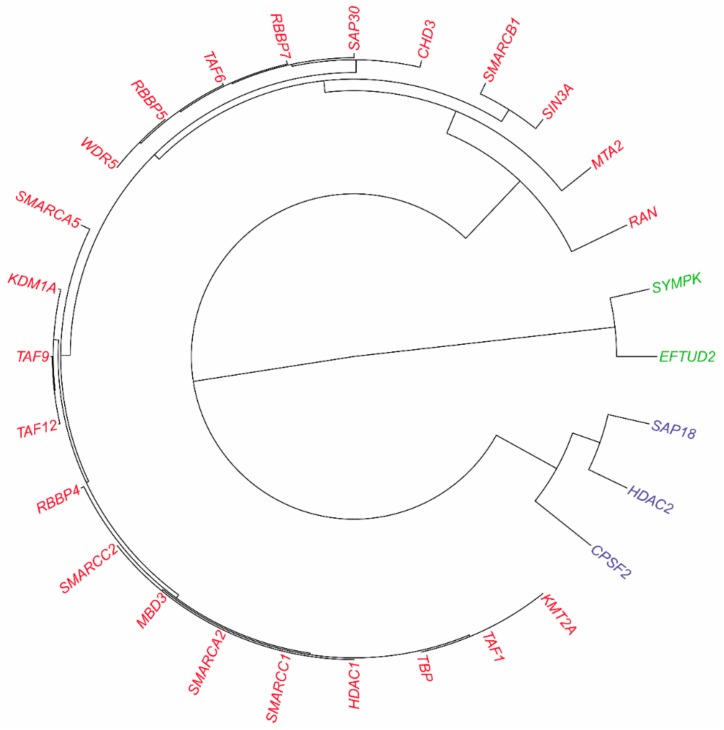
Predicted physical interactions via supervised learning and inferred hierarchical sub-complexes via GO semantic similarity based functional clustering within ALL-1 supercomplex from CORUM [4] (28 subunits).

As illustrated in Figure 3A, about 57% of the complexes from CORUM [4] were predicted to have their subunits fully connected (i.e., connection degree equal to or extremely close to one). For these complexes, topological clustering was not applicable and GO semantic similarity based functional clustering was used instead to infer the inner substructures. Take ALL-1 supercomplex for an example. The complex was composed of 28 subunits with predicted connection degree equal to 95.77%. ALL-1 is a histone methyltransferase that assembles a supercomplex to get involved in transcriptional regulation [31]. Most subunits of the supercomplex are components of human transcription complexes TFIID (including TBP), SWI/SNF, NuRD, hSNF2H and Sin3A. The other subunits are involved in RNA processing or in histone methylation. If we roughly decomposed ALL-1 complex into three sub-complexes, functional clustering inferred the hierarchically organized sub-complexes as shown in Figure 6.

**Figure 7:**
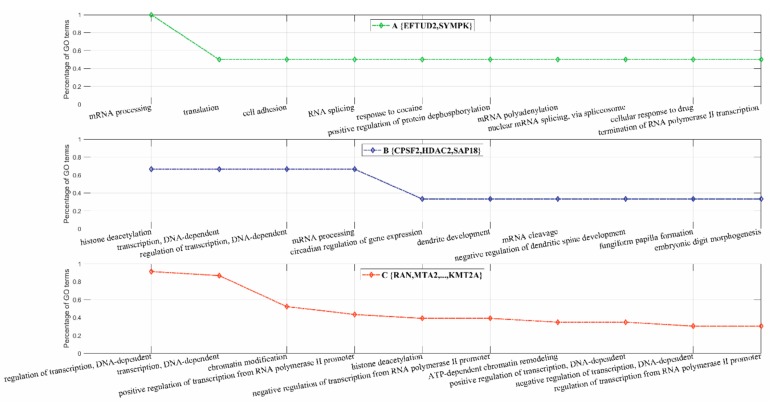
GO enrichment analysis of the GO semantically inferred sub-complexes within complete ALL-1 supercomplex (28 subunits). **A**. The sub-complex {EFTUD2,SYMPK}. **B**. The sub-complex {CPSF2,HDAC2,SAP18}. **C**. The sub-complex {RAN, MTA2, …, KMT2A}.

Further GO enrichment analyses of the sub-complexes within ALL-1 supercomplex are illustrated in Figure 7A–C. The sub-complex {EFTUD2, SYMPK} was mainly involved in the processes of mRNA processing (GO:0006397), translation (GO:0006412), nuclear mRNA splicing via spliceosome (GO:0000398), etc. (see Figure 7A). The sub-complex {CPSF2, HDAC2, SAP18} was mainly involved in the processes of histone deacetylation (GO:0016575), regulation of transcription DNA-dependent (GO:0006355), mRNA processing (GO:0006397), dendrite development (GO:0016358), etc. (see Figure 7B). The last sub-complex was mainly involved in regulation of transcription (e.g., GO:0006355 regulation of transcription, DNA-dependent; GO:0016568 chromatin modification; GO:0045944 positive regulation of transcription from RNA polymerase II promoter, etc.) (see Figure 7C).

### 3.3. Comparison with the Related Work

#### 3.3.1. Predicting Physical Interactions within Complexes

To our knowledge, there are only two studies on inferring direct contacts and substructures within complexes [11,12]. Both methods first predict physical subunit interactions within complexes. Different from this proposed framework, the two methods [11,12] use the interactome-scale physical protein–protein interactions as positive training data to reconstruct genome-scale physical PPIs, which are further mapped into complexes to infer direct contacts between subunits. However, the patterns of direct and indirect interactions within complexes are potentially quite different. In this proposed framework, the direct and indirect interactions in the training data were both restricted within complexes, so that the trained model was more biologically sound and interpretable.

The two methods [11,12] do not provide the performance metrics of cross validation such as precision, recall, MCC and AUC scores. Furthermore, they neither provide the performance of independent test. Friedel et al. [12] report 49.1% true positive rate at 13.6% false positive rate. As shown in Figure 2A, the proposed framework achieved nearly 80% true positive rate at 13.6% false positive rate. This result showed that the proposed framework outperformed the related work in identifying direct contacts within complexes.

#### 3.3.2. Inferring Substructures within Complexes

The two related studies [11,12] divide the direct-contact subunits into sub-complexes without considering the hierarchical or overlap substructures within complexes. Similar to complexes identification, sub-complexes discovery also needs sophisticated graph clustering techniques. For the fully-connected complexes with connection degrees equal to or very close to one, the two related studies [11,12] cannot identify the inner substructures, but this proposed framework explicitly solved the problem via GO semantic similarity based functional clustering. To our knowledge, no experimentally verified sub-complexes are available to evaluate the performance of the proposed framework and related methods.

Nevertheless, we still compared the maximum modularity clustering method (MMC) [17] used by this proposed framework with the well-accepted Markov clustering (MCL) method [16] on the complexes from CORUM [4] and HPRD [16]. We first binarized the complexes from CORUM [4] and HPRD [5] into co-complex networks and then compared MMC with MCL to find out which method could best recover the known complexes from the co-complex networks. As shown in Table 2, 11.71% and 11.78% of the reference complexes from CORUM [4] and HPRD [5] were exactly predicted by MMC [17] (ξ=1, recall metric), respectively; and 32.57% and 16.67% of the predicted clusters exactly matched the reference complexes from CORUM [4] and HPRD [5] (ξ=1, precision metric), respectively. However, MCL [14] at most predicted 1.16% of the reference complexes from CORUM [4] and HPRD [5] and yielded a large number of singleton clusters accounting for at least 50% of the entire clusters.

If the Jaccard index threshold ξ was set 0.5, 52.34% and 54.26% of the reference complexes from CORUM [4] and HPRD [5] matched the predicted clusters (ξ=0.5, recall metric), respectively; and 80.99% and 77.68% of the predicted clusters matched the reference complexes from CORUM [4] and HPRD [5] (ξ=0.5, precision metric), respectively. These results showed that the MMC method [17] excelled the commonly-used MCL method [16] and was a good solution to identifying substructures within complexes.

## 4. Discussion

A fine-grained resolution of direct subunit contacts and inner substructures within complexes is significant to understanding how complexes work. To the best of our knowledge, there are very few computational studies on predicting substructures within complexes to date. The two related studies [11,12] use the interactome-scale physical protein–protein interactions (PPI) as training data to predict genome-scale physical PPIs, which are further mapped into complexes to infer direct subunit contacts. However, the interactome-scale and complexes-scale physical PPI patterns are potentially quite different. In this study, we proposed a computational framework to learn the patterns of direct and indirect subunit interactions within complexes and further identified the inner substructures via graph and functional clustering. The sampling of direct and indirect PPIs was restricted within complexes to train an l_2_-regularized logistic regression model. The computational results of cross validation and independent test show that the proposed framework outperformed the related methods in terms of predicting direct subunit contacts within complexes.

Topological clustering of directly-contacted subunits requires sophisticated graph clustering techniques to infer the hierarchical and overlap substructures within complexes. In this study, we employed the maximum modularity clustering method (MMC) to infer sub-complexes from the predicted networks of physical subunit interaction. For the fully-connected complexes, we used GO semantic similarity based functional clustering to infer the inner substructures. As compared to the related studies, this proposed framework demonstrated two major advantages. First, the sampling of direct and indirect subunit interactions was restricted within complexes, so that the trained model was more biologically interpretable. Second, we used MMC method and functional clustering method to infer the hierarchical and overlap substructures within partially-connected and fully-connected complexes, respectively. The predicted direct contacts and substructures within complexes potentially provide valuable clues for future biomedical research.

## Figures and Tables

**Table 1:** Performance of cross validation and independent test.

Cross Validation	Size	Combined-instance			Homolog-instance			Target-instance		
		PR	SE	MCC	PR	SE	MCC	PR	SE	MCC
Direct contact	9125	0.8553	0.8830	0.7627	0.8554	0.8830	0.7629	0.8613	0.8830	0.7673
Indirect contact	9125	0.8790	0.8506	0.7611	0.8792	0.8508	0.7613	0.8786	0.8562	0.7655
(Acc; MCC)		(86.68%; 0.7616)			(86.69%; 0.7618)			(86.96%; 0.7663)		
(ROC-AUC)		(0.9308)			(0.9309)			(0.9320)		
F1 Score		0.8740			0.8690			0.8720		
Independent test		HPRD			CORUM			KEGG		
		(+83.99%; −86.38%)			(+83.10%; −86.26%)			( ; −75.00%)		

Note: sign + denotes positive recall/recognition rate and sign – denotes negative recall/recognition rate.

**Table 2:** Graph clustering performance on CORUM [4] and HPRD [5].

	Exact Match (ξ=1)			Match (ξ=0.5)		
	Precision	Recall	F-score	Precision	Recall	F-score
**CORUM**	0.3257	0.1171	0.2294	0.8099	0.5234	0.6359
**HPRD**	0.1667	0.1178	0.1381	0.7768	0.5426	0.6389

## Supplementary Materials

The following are available online at https://www.mdpi.com/2218-273X/9/11/656/s1, Supplementary Files S1–S3: positive and negative training data.
